# Gastrointestinal endoscopy in early diagnosis and treatment of gastrointestinal tumors

**DOI:** 10.12669/pjms.36.2.707

**Published:** 2020

**Authors:** Chunmei Li, Lingzhi Li, Juan Shi

**Affiliations:** 1 Chunmei Li, Digestive Endoscopy Center, Binzhou People’s Hospital, Shandong, 256610, China; 2 Lingzhi Li, Outpatient Department, Binzhou People’s Hospital, Shandong, 256610, China; 3 Juan Shi, Department of Cardiothoracic Surgery, Binzhou People’s Hospital, Shandong, 256610, China

**Keywords:** Gastrointestinal tumors, Gastrointestinal endoscopy, Diagnostic value

## Abstract

**Objective:**

To explore the value of gastrointestinal endoscopy in the early diagnosis and treatment of gastrointestinal tumors and lay a foundation for the diagnosis and treatment of gastrointestinal tumors.

**Methods:**

One hundred and eight patients with gastrointestinal tumors who were admitted to our hospital from August 2016 to April 2018 were retrospectively analyzed and divided into observation group and control group according to different diagnostic methods, 54 cases in each group. The control group was treated with traditional endoscopy (white light imaging) and traditional surgery, while the observation group underwent narrow band imaging (NBI) based on endoscopic examination and endoscopic mucosal resection. The image quality scores (morphological image, gastric pit image and capillary image), diagnostic accuracy, surgery related clinical indicators (operation time, intraoperative bleeding volume, hospitalization days) and complications were observed and compared between the two groups.

**Results:**

The morphological image, gastric pit image and capillary image scores of the observation group were higher than those of the control group (P<0.05). The diagnostic accuracy rate of the observation group was 96.30%, which was significantly higher than 75.93% (P<0.05). The operation time and hospitalization days of the observation group were shorter than those of the control group, and the intraoperative bleeding volume of the observation group was less than that of the control group; the differences were statistically significant (P<0.05). The incidence of complications of the observation group was lower than that of the control group, and the difference was statistically significant (P<0.05).

**Conclusion:**

Gastrointestinal endoscopy can accurately identify the pathological changes of tumors in the early diagnosis and treatment of gastrointestinal tumors, improve the diagnostic accuracy rate, and guide the implementation of treatment measures to improve clinical indicators. Moreover the incidence of postoperative complications is low. It is worth clinical promotion.

## INTRODUCTION

Gastrointestinal tumor, a kind of common malignant tumor in clinics, includes gastric tumor, esophageal tumor rectal tumor, colon tumor, etc.^1^,^2^ In recent years, due to the influence of different factors, the incidence of digestive tract malignant tumors in China is increasing, seriously endangering the lives and health of patients.^3^ As the digestive tract involves many parts such as stomach, intestine, esophagus, liver and gallbladder, pancreas, etc., it is very vulnerable to bacterial and viral infections, causing local inflammatory lesions or cancers, and moreover its hidden location may lead to missed diagnosis and delay treatment.^4^,^5^ Most patients with digestive tract tumors are diagnosed at advanced stage; hence they have short survival time and poor prognosis.^6^ Therefore, early diagnosis of digestive tract tumors is extremely important.

In recent years, with the continuous development of medical technology, new gastrointestinal endoscopy technology has been widely used in clinical practice. Digestive endoscopy has gradually evolved from a traditional diagnostic tool to a diagnostic and therapeutic tool.^7^ Studies show that narrow band imaging (NBI) magnifying endoscopy could clearly observe the fine structure of the digestive tract to make early cancer in the digestive tract being diagnosed effectively and treated by minimally invasive treatment.^8^,^9^ In order to explore the application value of digestive endoscopy in early gastrointestinal tumor, 54 patients with early gastrointestinal tumors who were diagnosed and treated by NBI endoscopy in our hospital were selected as observation group, and 54 patients with early gastrointestinal tumor who were diagnosed by traditional endoscopy (white light imaging) and treated by conventional surgery in the same period were selected as control group, and the diagnosis and treatment effect of the two groups were compared.

## METHODS

One hundred and eight patients with gastrointestinal tract tumors who were admitted to our hospital from August 2016 to April 2018 were retrospectively analyzed. The inclusion criteria were: the clinical symptoms and pathological examination results of the patients both satisfying the relevant diagnostic criteria of gastrointestinal tumors,^10^ being diagnosed as early gastrointestinal tumors by computed tomography (CT), having no dysfunction in the kidney, liver and heart and mental diseases, and having clear consciousness. All the patients were divided into an observation group and a control group according to diagnostic methods. In the control group, 29 cases were male, 25 cases were female; they aged (59.2±6.8) year and had a disease course of (4.3±1.6) months. As to types of tumors, there were 12 cases of early esophageal tumor, 16 cases of early gastric tumor and 26 cases of early colorectal tumor. In the observation group, there were 28 males and 26 females; they aged (58.1±6.3) years and had a disease course of (4.5±1.4) months. As to types of tumors, there were 13 cases of early esophageal tumor, 17 cases of early gastric tumor and 24 cases of early colorectal tumor. There was no significant difference in baseline data such as sex, age, course of disease, type of tumor, etc. between the two groups. This study was approved by the ethics committee (Ref.No. 111, Dated on October 20, 2018) of our hospital, and all the selected patients signed the informed consent.

### Diagnosis and treatment

The control group was treated with traditional endoscopy (white light imaging) and traditional surgery. White light endoscopy was used to perform the corresponding examination. If lesions were found, the lesions were taken out immediately, and then pathological examination was carried out. For patients who were definitely diagnosed having gastrointestinal tumors, traditional surgery was used.

The observation group was given NBI endoscopy and endoscopic mucosal resection. The patients orally took surface anaesthetic and were assisted to take a lateral position. The NBI endoscope was inserted to the digestive tract to clearly observe the morphology of micro glandular tube and capillaries and infiltration depth of pathological tissues, and the degree of pathological changes was evaluated. Then they were intravenously anesthetized. Under the guidance of endoscope, the pathological tissues of the patients were processed by staining (the stained area was 2 mm larger than the area of the pathological tissues), and the staining boundary was marked. 2 ~ 3 ml of indigo carmine and adrenaline saline (1:10000) were injected at the submucosal layer of the pathological tissues. After the lifting sign of the pathological tissues became positive, the pathological tissues were resected completely using electrocoagulation, and the resected pathological tissues were sent to the department of pathology for pathological examination.

### Observation indicators

The image quality scores (morphological image, gastric pit image and capillary image) were observed and compared.

### Statistical analysis

SPSS 21.0 was used for data processing. The counting data were expressed as % and the comparison was performed by X^2^ test. The measurement data were expressed as Mean±SD and the paired t test was used for comparison between the two groups. The difference was thought statistically significant if P<0.05.

## RESULTS

### Comparisons of image quality scores between the two groups

By comparing the image quality scores of the two groups, it was found that the observation group was better than the control group, and the differences were statistically significant (P<0.05, Table-I).

**Table-I:** Image quality scores of two groups of patients with diagnostic methods.

Group	Morphological image	Gastric pit image	Capillary image
Observation group	3.94±0.77	3.78±0.86	3.55±0.84
Control group	2.31±0.63	1.96±0.53	2.26±0.70
t	11.976	13.475	8.751
P	<0.05	<0.05	<0.05

### Comparison of diagnostic methods between two groups

**Fig.1:**
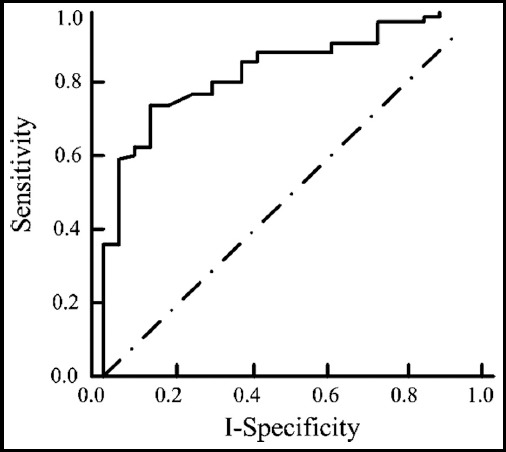
ROC curve for diagnosis of digestive tract tumors in control group.

**Fig.2:**
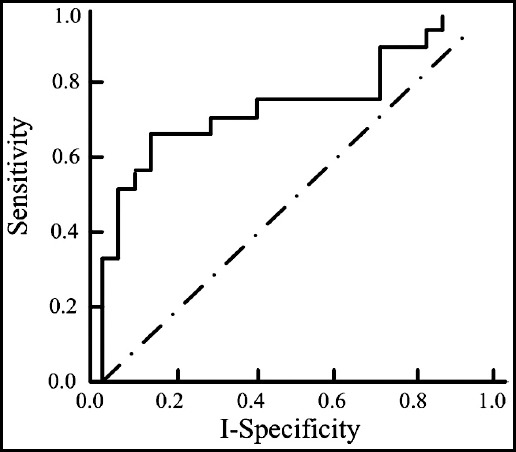
ROC curve for diagnosis of digestive tract tumors in observation group.

The area under the receiver operating characteristic (ROC) curve of the observation group was 0.808 [95% CI (1.057, 4.221)], and the diagnostic accuracy was 80.0% (36/45). The area under the ROC curve of the control group was 0.974 [95% CI (0.806, 2.622)], and the diagnostic accuracy was 55.0 (22/40). The diagnostic accuracy of digestive tract tumors in the observation group was higher than that in the control group (P<0.05, Table-II, Fig.1 and 2).

**Table-II:** Diagnostic accuracy between two groups.

Group	Early esophageal cancer	Early gastric cancer	Early colorectal cancer	Diagnostic accuracy
Observation group	12(92.31)	17(100)	23(95.83)	52(96.30)
Control group	9(75)	13(81.25)	19(73.08)	41(75.93)
X^2^				7.570
P				<0.05

### Comparison of incidence of complications between the two groups

The incidence of complications in the observation group was 9.26% (5/54), including one case of bleeding, two cases of abdominal pain, one case of diarrhea and one case of nausea and vomiting. The incidence of complications in the control group was 25.93% (14/54), including five cases of abdominal pain, four cases of diarrhea and five cases of nausea and vomiting. The incidence of complications in the observation group was lower than that in the control group (X^2^=4.357, P<0.05).

## DISCUSSION

Gastrointestinal tumor is a common disease of the digestive system, and its mortality rate is high in the world. In China, the mortality rate caused by gastric cancer ranks third among malignant tumors, while colorectal cancer ranks fifth. It is generally acknowledged that early diagnosis and systematic treatment of tumors can effectively curb the spread of tumors and even cure tumors.^11^,^12^ Therefore, early detection, diagnosis and treatment are crucial to improve the prognosis of patients with digestive tract tumor.

In the past, white-light endoscopy has been widely used in clinical diagnosis of digestive tract tumors. With the development of medical devices and the development of imaging diagnosis technology, the diagnostic technology schemes for clinical use are becoming more and more perfect. With the support of microelectronics, computer, ultrasound and other technologies, digestive endoscopy technology which is based on traditional endoscopy technology tends to have wider scope of observation and higher image resolution and becomes highly operable.^13^ At present, narrow-band endoscopic imaging and endoscopic mucosal resection are widely used in the diagnosis of early gastrointestinal tumors, which greatly improves the diagnosis and treatment of early gastrointestinal tumors.^14^ The application of narrow-band imaging technology in early diagnosis of digestive tract tumors can effectively enhance the contrast effect of uncolored mucosa, and under the assistance of special narrowband filter, the internal condition of digestive tract can be clearly observed and the slight changes in digestive tract mucosa can be displayed, which significantly improve the diagnostic accuracy.^15^

In addition, in the treatment of digestive tract tumors, corresponding surgical treatment can rely on digestive endoscopy, which can not only reduce the surgical wounds of patients, but also will not cause significant damage to the surrounding organs and effectively shorten the operation time and hospitalization time.^16^,^17^ The research results of Liang showed that the accuracy of digestive endoscopy in the diagnosis of early gastrointestinal cancer was 97.5%,^18^ the operation time and hospitalization time of patients in observation group were significantly shorter than those in control group, the amount of bleeding during operation was significantly less than that in control group, the incision of operation was smaller than that in control group, the incidence of complications in observation group was significantly lower than that in control group, and the mental state score of the observation group was significantly higher than that of the control group; in conclusion, digestive endoscopy technology has a significant efficacy in the diagnosis and treatment of early gastrointestinal tumor, with high accuracy and safety.

The ROC curve analysis showed that both traditional endoscopy and digestive endoscopy could effectively diagnose digestive tract tumors, but 52 cases were detected in the observation group and 41 cases in the control group. The diagnostic accuracy of the observation group for early gastrointestinal tumors was significantly higher than that of the control group. It was concluded that digestive endoscopy could effectively improve the detection rate of early gastrointestinal tumors, providing a reference for the correct diagnosis of tumors. In addition, angiogenesis is an important indicator to determine the formation of early gastrointestinal tumors; hence it can effectively diagnose early gastrointestinal cancer by observing the vascular characteristics. In this study,^19^ it was found that narrow-band imaging endoscopy had significantly higher scores of morphological images, gastric pit images and capillary images than conventional endoscopy. Traditional laparotomy has large incision, great damage to the patients’ body, large wound in contact with air, and high risks of infection and other complications after operation in the treatment of early gastrointestinal tumors, which is not conducive to postoperative recovery and prolonged hospitalization time.^20^ Endoscopic mucosal resection is a kind of minimally invasive surgery, which has a high resection rate for the lesion tissue, relatively less trauma to the patients’ body, and less pain for patients; hence it is conducive to the recovery of patients after surgery.^21^ Moreover, the incision is small and the probability of complications is low. In this study, the operation time, incidence of complications and hospitalization time of endoscopic resection were better than those of traditional laparotomy, which was consistent with the results of Tang et al.^22^

**Table-III:** QLQ-C30 scale score between two groups.

Group	Operation time (min)	Intraoperative bleeding volume (mL)	Hospitalization days (d)
Observation group	70.6±14.1	118.8±19.4	7.6±2.2
Control group	81.2±17.5	82.4±1.6	4.2±1.1
t	4.145	10.987	9.102
P	<0.05	<0.05	<0.05

## CONCLUSION

In conclusion, the application of digestive endoscopy in the early diagnosis and treatment of digestive tract tumors can accurately identify tumors, effectively improve the image quality, reduce the risk of misdiagnosis and missed diagnosis, shorten the operation time, and reduce the damage to the body and the incidence of complications after operation, which is of high clinical value.

### Authors’ Contribution:

**CML & LZL:** Study design, data collection and analysis.

**LZL & JS:** Manuscript preparation, drafting and revising.

**CML & JS:** Review and final approval of manuscript, are responsible for integrity of research.
